# Transgenic Expression of the Amyloid-β Precursor Protein-Intracellular Domain Does Not Induce Alzheimer's Disease–Like Traits *In Vivo*

**DOI:** 10.1371/journal.pone.0011609

**Published:** 2010-07-16

**Authors:** Luca Giliberto, Cristina d'Abramo, Christopher Michael Acker, Peter Davies, Luciano D'Adamio

**Affiliations:** 1 The Litwin-Zucker Research Center for the Study of Alzheimer's Disease, The Feinstein Institute for Medical Research, North Shore–Long Island Jewish Health System (North Shore-LIJ), Manhasset, New York, United States of America; 2 Department of Pathology, Albert Einstein College of Medicine, New York, New York, United States of America; 3 Department of Microbiology and Immunology, Albert Einstein College of Medicine, New York, New York, United States of America; Case Western Reserve University, United States of America

## Abstract

**Background:**

Regulated intramembranous proteolysis of the amyloid-β precursor protein by the γ-secretase yields amyloid-β, which is the major component of the amyloid plaques found in Alzheimer's disease (AD), and the APP intracellular domain (AID). *In vitro* studies have involved AID in apoptosis and gene transcription. *In vivo* studies, which utilize transgenic mice expressing AID in the forebrain, only support a role for AID in apoptosis but not gene transcription.

**Methodology/Principal Findings:**

Here, we have further characterized several lines of AID transgenic mice by crossing them with human Tau-bearing mice, to determine whether over-expression of AID in the forebrain provokes AD-like pathologic features in this background. We have found no evidence that AID overexpression induces AD-like characteristics, such as activation of GSK-3β, hyperphosphorylation of Tau and formation of neurofibrillary pathology.

**Conclusions/Significance:**

Overall, these data suggest that AID transgenic mice do not represent a model that reproduces the overt biochemical and anatomo-pathologic lesions observed in AD patients. They can still be a valuable tool to understand the role of AID in enhancing the cell sensitivity to apoptotic stimuli, whose pathways still need to be characterized.

## Introduction

Alzheimer disease (AD) is characterized by the production of amyloidogenic peptides, neurofibrillary tangles (NFT) and neurodegeneration [1], [2]. The prevailing pathogenic theory, the “Amyloid Cascade Hypothesis” [3], posits that the accumulation of neurotoxic amyloidogenic peptides triggers Tauopathy, neurodegeneration, cognitive and behavioral changes. In AD, the amyloid lesions are formed by Aβ42, which derives from the Amyloid-β Precursor Protein (APP). APP is cleaved by β-secretase to releases the soluble β-ectodomain (sAPPβ) and the membrane-bound COOH-terminal fragment C99. C99 is in turn processed by γ-secretase to produce the APP intracellular domain (AID/AICD) and Aβ peptides. More recently, attention of researchers in the field has shifted from Amyloid plaques to Aβ42 oligomers as the main cause of AD [4], [5], [6]. However, the amyloid cascade hypothesis is yet to be validated, and causes of dementia may be multifaceted and involve other mechanisms. Several investigators have suggested that other APP-derived fragments may cause or contribute to AD pathogenesis. Evidence hints to specific functions and disfunctions for holoAPP and APP-derived polypeptides. An APP fragment derived from sAPPβ interacts with DR6 to trigger axon pruning and neuron death [7]. The short AID/AICD is a biologically active intracellular peptide, which modulates cell death, gene transcription and Ca^++^ homeostasis [8], [9], [10], [11], [12], [13], [14], [15], [16], [17], [18], [19]. Caspase-derived APP fragments, such as C31 [20] and Jcasp [9], [21], posses *in vitro* toxic activities. Because of these evidence, various APP-derived fragments, such as AID/AICD [10], [22], C31 [23], JCasp [9], sAPPβ [7] have been implicated in neurodegenerative processes.

Mouse models are critical to explore both pathogenesis and therapy of human diseases. All animal models used to study human neurodegenerative diseases consist of transgenic mice carrying mutant forms of genes shown to be involved in human dementia [24], [25], [26]. A serious limitation of these models is that their design is predicated on the assumption that development of amyloid plaques and neurofibrillary tangles is directly related to the cognitive and behavioral changes associated with human dementia. To determine whether AID could trigger an AD-like neurodegenerative process *in vivo*, we have made transgenic mice expressing AID-peptides in the CNS [27]. A previous analysis of these mice showed that AID can modulate apoptosis *in vivo*. However, a role of AID in gene transcription *in vivo* [28] could not be confirmed. Here, we have further characterized our AID transgenic mice to determine whether over-expression of AID in the forebrain, when human Tau is also expressed, provokes AD-like features, as recently suggested [22]. To this end, we have crossed our AID lines with hTau mice [29], [30] overexpressing human Tau. We have chosen this model as it shows progressive, age-related, Tau pathology in forebrain regions of the brain which are also affected in human AD pathology (hippocampus, parahippocampal cortex, frontal cortex etc.) and which overlap with the pattern of expression of the AID transgene, without necessarily expressing mutated Tau.

## Materials and Methods

### Ethics Statement

Mice were handled according to the Ethical Guidelines for Treatment of Laboratory Animals of Albert Einstein College of Medicine. The procedures were described and approved in animal protocol number 20040707.

### AID mice generation and crossing with hTau mice to yield AID/hTau transgenic mice

AID mice were generated as described before [31]. Briefly, the cDNA sequences corresponding to AID 50, 57 or 59, were subcloned into the pNN vector, downstream of the 8Kb CamKIIα promoter, driving the espression of the transgene in the forebrain of the postnatal mouse [32]. Transgenic mice, initially on a FVB background, were backcrosses, at least for 8 generations, onto the C57Bl/6 background. hTau mice were obtained as described [29] by crossing 8c mice, expressing a human Tau transgene, H1 haplotype driven by the Tau promoter [33], with Tau knock-out (KO) mice that have a targeted disruption of exon 1 of Tau [34]. Animals were backcrossed 10 times to C57BL/6J background. AID transgenic mice were crossed with hTau mice to yield mice expressing AID transgene, human Tau transgene and one allele copy of mouse Tau.

### Monoclonal antibodies specific for Tau and Tau phosphorylations

The following monoclonal antibodies have been produced as described earlier [35].

DA9 (IgG1): total Tau aa102–140; TG5 (IgG1): total Tau aa220–240 (murine and human); CP27 (Ig2B): total Tau aa130–170 (human specific); CP13 (IgG1): pSer202; PHF1 (IgG1): pSer396/Ser404; MC1 (IgG1): N-terminal conformational change, Exon 10; CP17 (IgG3): pThr231; CP9 (IgM): pThr231. Antibodies were used pure (hybridoma medium), purified or biotinylated. Biotinylation of antibodies was performed using EZ-Link NHS-PEO Solid Phase Biotinylation Kit (Pierce, # 21440).

### Heat stable preparation for Tau

Mice were sacrificed by cervical dislocation after Isoflurane anesthesia. One of the two brain hemispheres was processed for protein and Tau extraction, the other for immunohistochemistry. Forebrains were homogenized in 400µL/100mg of Homogenizing Buffer (TBS-10mM TRIS, 140mM NaCl, pH 7.4-, Roche Complete-EDTA Protease Inhibitor, 1mM PMSF, 1mM Na3VO4, 10mM NaF, 2mM EGTA) for 20″ with a Polytron homogenizer, on ice. For heat stable Tau, to a 500µL aliquot of homogenate, NaCl was added to 250mM, and βME was added to 5% final concentrations, and the mix vortexed. Homogenates were heated at 100°C for 15′, mixed again by vortexing and cooled in ice for 30′, vortexed briefly again and spun down at 20,000g for 10′ at 4°C. The supernatant, containing heat stable microtuble protein enriched for Tau, was retained and used for subsequent experiments. Protein concentration was measured in the original homogenate in order to load equal amounts of protein for western blot and ELISA.

### ELISA

Clear, flat bottom, 96 well plates were coated overnight with an antibody specific for total Tau (DA9) 2µg/mL in Coating Buffer (230mM K2HPO4, 135mM KH2PO4, 130mM NaCl, 2mM EDTA, 0.05% NaN3, pH 7.2). Wells were blocked with 100% Starting Block (Pierce) for 1 hour at room temperature. Homogenates were then added: brain homogenates, prepared as described above, were mixed 1/1 with 0.1% SDS, briefly vortexed and spun for 10′ at 20,000g, at room temperature, and the supernatant loaded on the ELISA plate at 1/250 to 1/2000 dilution in 20% Superblock-TBS (Pierce) as needed, and left overnight, at 4°C, gently shaking. Purified primary antibodies we added to the plate, diluted in 20% Superblock-TBS, as follow: TG5-biotinylated 1/500000, CP27 (Ig2B) 1/20000, CP13 (IgG1) 1/20000, PHF1-biotinylated 1/10000, CP17-biotinylated 1/20000, MC1 (IgG1) 1/20000, and left for 2 hours at room temperature, gently shaking. Secondary antibodies, Streptavidin-HRP or HRP-Goat-anti mouse IgG2B/IgG1, 1/5000 in 20% Superblock-TBS (Pierce), were left on the plate for 1 hour as above. Fifty µL of TMB (Pierce) were added and left for 20′, room temperature, light shielded, gently shaking, and the reaction was stopped with same volume of 2M Sulfuric Acid, and read at 450nM with an automated Tecan plate reader. Every step was followed by 5 automated washes with washing buffer (100mM NaCl, 10mM Tris Base, 0.1% Tween20).

### Western Blot

For Tau, heat stable preparations were run on a 4–12% tris-HCl precast gel (Biorad), blotted on a 0.45µM Nitrocellulose membrane and probed with monoclonal antibodies specific for total Tau or site specific Tau phosphorylations as follows: DA9 (1/3000), CP13 (1/3000CP9 (1/3000), PHF1 (1/3000), MC1 (1/500). Antibodies were diluted in 5% milk in TBS. Western Blots were developed either with the ECL system on film or by means of 4-Chloro-Naftol reaction on the membranes. For GSK, brains were homogenized as described for ELISA, subjected to PAGE as described above, and the membranes were probed with antibodies against GSK3α/β (Santa Cruz SC-7291, 1/1000), GSK3βpSer9 (Cell Signaling #9323, 1/1000), GSK3α pTyrα279/β216 (Invitrogen #44604G), diluted in 20% Superblock-TBS, and anti β-Actin (Sigma-Aldrich A1978) 1/8000 in 5% Milk-TBS-0.1%Tween20.

### Immunohistochemistry

Brains were fixed in 4% PFA overnight and cut sagittally with a vibratome the next day. Selection of corresponding sections from each mouse was performed as follows: brains were cut sagittally at 50µM thickness, from the lateral to the medial aspect, preserving the cerebellum. For each antibody, 3 sections were stained, starting at section #15 (i.e. 750µM form the lateral pole), and taking every 7^th^ section inward (i.e. 350µM apart from each other). Free-floating sections were conserved in TBS (50mM Tris, 150mM NaCl, pH 7.6)/0.02%NaN3, and the staining was performed on multiwell plates. Endogenous peroxidases were quenched with 3% H2O2/0.25% Triton X-100/TBS for 30′. Non-specific binding was blocked with 5% Milk-TBS for 1 hour at room temperature. Primary antibodies are as follows: NeuN (Invitrogen #187373, 1/1000), IbA1 (WAKO #019-19741, 1/1500), and non-purified anti Tau antibodies DA9 (1/5000), CP13 (1/5000), PHF1 (1/5000), MC1 (1/200), all diluted in 5% Milk-TBS, and left overnight at 4°C, shaking. Biotin-conjugated secondary antibodies directed against the specific isotypes were diluted 1/1000 in 20% Superblock, left for 2 hours at room temperature, and likely Streptavidin-HRP was incubated for 1 hour. Staining was visualized by 3,3′-Diaminobenzidine. Each step was followed by 5×5′ washes in TBS.

### Statistical analysis

All quantified data represent an average of at least triplicate samples. Error bars represent standard errors of the mean. Statistical analysis was done by 2-Way ANOVA and Bonferroni Post Test. Significance was determined by Student's t test and a p<0.05 was considered significant.

## Results

### AID/hTau mice general characteristics

Mice are presenting with normal growth and weaning, thrive at appropriate age. No significant differences were observed in breeding and litter size and survival compared to C57BL/6 mice. Up to 21 months of age, there is no prevalent pathology, susceptibility to infections, and animals appear active and alert.

### Tau phosphorylation and brain load are not univocally affected by AID overexpression

**Figure 1:**
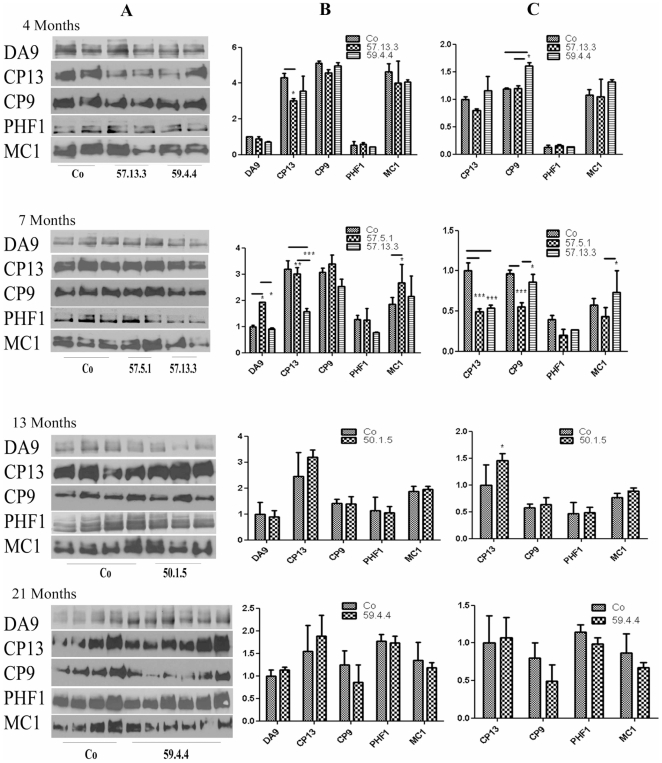
Western Blot analysis of Tau phosphorylation. Heat Stable Preparation of Tau, from forebrains of AID/hTau mice, were run on PAGE (Column A) and probed with antibodies specific for total Tau (DA9), pSer202 (CP13), pThr231 (CP9), pSer396/Ser404 (PHF1) and conformational modification of human Tau (MC1). Densitometric quantification, both raw (Column B) and over total Tau (DA9, Column C), shows no clear cut change in the pattern of Tau phosphorylation due to AID overexpression, related neither to AID length, mouse line nor age. Experiments were repeated at least 3 times on at least 2 mice/line. Quantifications units are arbitrary. *p<0.05; **p<0.01; ***P<0.001.

**Figure 2:**
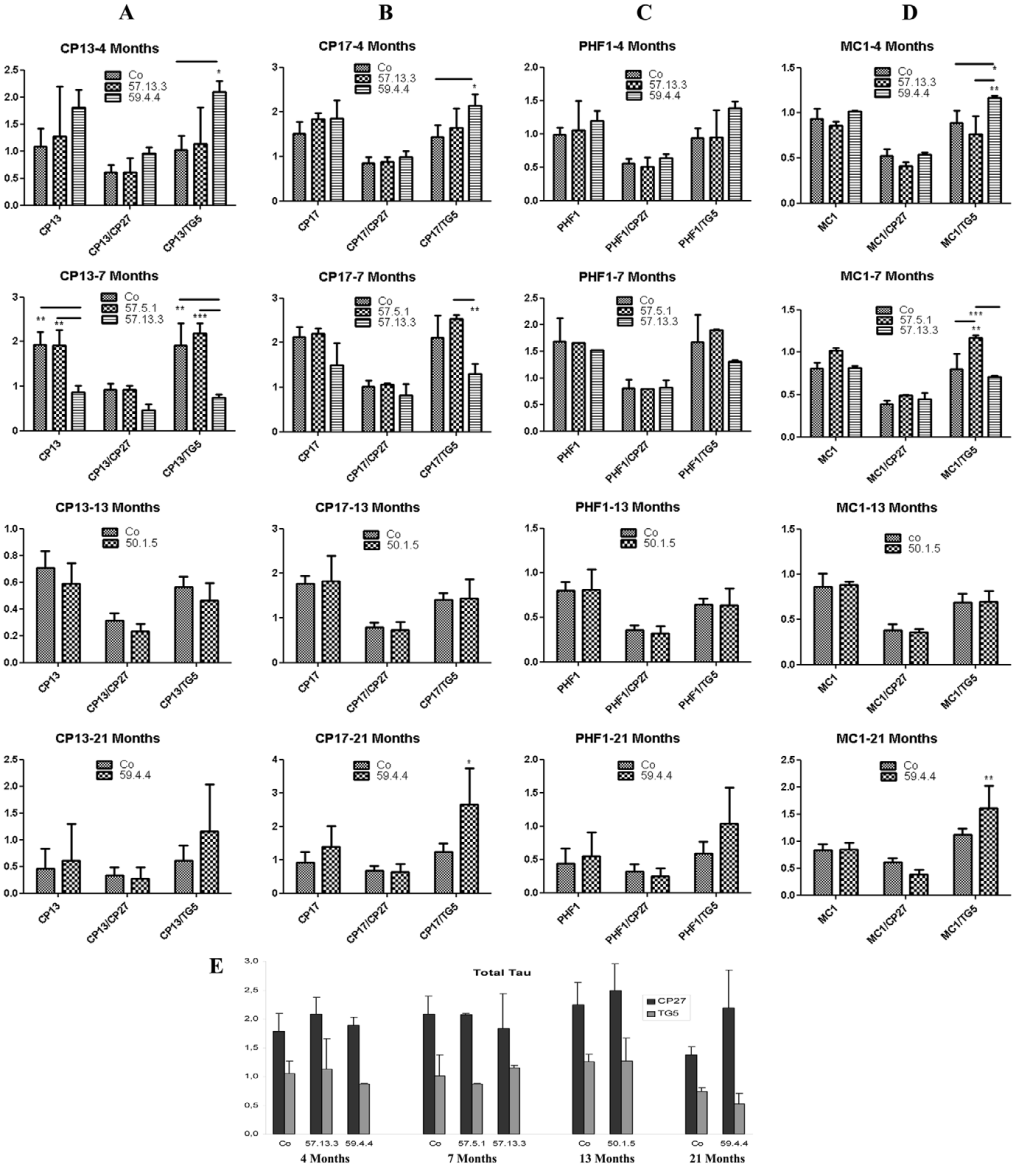
ELISA analysis of Tau phosphorylation. Homogenate from forebrains of AID/hTau mice were anaylzed by mean of sandwich ELISA, capturing with the total Tau DA9 antibody, and revealing with antibodies specific for total human Tau (CP27), total mouse and human Tau (TG5), or for several Tau phosphorylations, pSer202 (CP13) (Column A), pThr231 (CP17) (Column B), pSer396/Ser404 (PHF1) (Column C) and conformational modification of human Tau (MC1) (column D). The top 16 panels show scattered differences in the phosphorylation pattern, which do not seem to be related to AID overexpression, length, mouse line or age. The bottom panel (E) shows how levels of total human and murine Tau are maintained in the different mouse lines and ages. Experiments were repeated at least 3 times on at least 2 mice/line. Quantifications units are arbitrary. *p<0.05; **p<0.01; ***P<0.001.

Tau accumulation, phosphorylation and axonopathy can be subtle and precede neuronal degeneration, tangle formation and cognitive or behavioural deficits [36], [37]. Thus, we have extensively searched for any contribution of AID overexpression to Tau pathology. We have used antibodies raised against specific Tau phosphorylation sites that characterize early (pSer202 and pThr231), or late (pSer396/404) stages of Tau phosphorylation in AD [37], [38]. In addition, we have also analyzed a typical conformational change in Tau that is found at the latest stages of AD, using the MC1 antibody. We have analyzed several mice bearing hTau and AID50, 57 or 59, corresponding respectively to the γ-secretase ε-cleavage and γ-cleavage producing Aβ42 or Aβ40 [27], at different ages, from 4 to 21 months. For each AID transgene, different lines derived from distinct founders were analyzed. Analysis of more than one AID50, 57 or 59 line is necessary to control for phenotypes dependent on the integration of the transgene in the mouse chromosomes, which can alter expression of endogenous mouse genes. As previously shown, these lines also expressed different levels of AID and they behaved differently as far as susceptibility to toxic stimuli *in vitro* [27]. In the western blot (WB) analysis (Fig. 1, Column A and quantified in Column B), total Tau (DA9) was not significantly and consistently altered in AID/hTau mice compared to hTau alone. Only line 57.5.1 showed an increase in total Tau at 7 months, but it was neither confirmed in line 57.13.3, nor by the ELISA analysis (Fig. 2, bottom panel), neither for human Tau alone, nor for human plus mouse Tau (CP27 vs TG5).

CP13 recognizes an early Tau phosphorylation epitope (pSer202). This phopshorylation was decreased in line 57.13.3 at 4 and 7 months on WB (Fig. 1, Column B), and also in line 57.5.1 when total Tau was accounted for (Fig. 1, Column C); contrarily, line 50.1.5 showed an increase of CP13 signal at 13 months (Fig. 1, Column C); ELISA data (Fig. 2) show no consistency in CP13 signal, that vary according to age and weather only human (CP27 weighed) or both human and murine (TG5 weighed) Tau are accounted for (Fig. 2, Column A). Thr231 phosphorylation (CP9) levels seem increased in line 59.4.4 at 4 months of age, but this increase is not maintained later in life in WB (Fig. 1, Column C). On the contrary, CP17 ELISA over total Tau shows an increase at all ages for AID59.4.4 (Fig. 2, Column B). Neither AID57 line shows consistent variation from controls at this site except a difference between the 2 AID57 lines at 7 months. Later phosphorylations (PHF1 Ser396/404) are not influenced by AID overexpression. Interestinlgy, on ELISA analysis, conformationally dependent MC1 signals show an increase in AID59 mice at 4 and 21 months (Fig. 2, Column D) when total Tau is considered. Less consistently, MC1 signal is augmented in the AID57 lines (Fig. 1, Column B and C; Fig. 2, Column D).

### Neuronal Tau distribution is not affected by AID overespression

**Figure 3:**
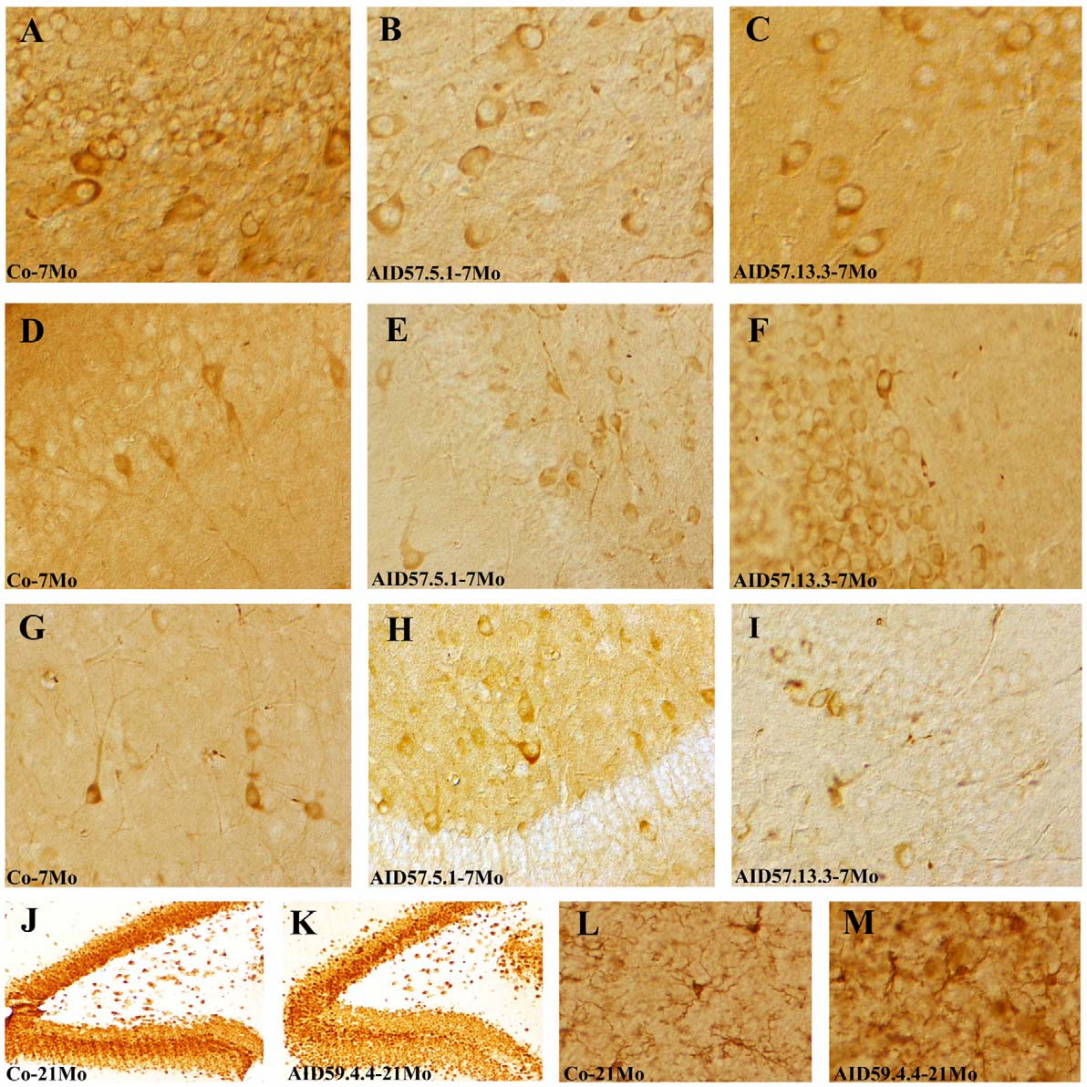
Immunohistochemical analysis of AID/hTau mice. Forebrains were stained with antibodies against total Tau (DA9, A, B, C), pSer202 (CP13; D, E, F), pSer396/404 (PHF1; G, H, I), the neuronal protein NeuN (J, K) and the microglia activation protein Iba1 (L, M). The expressed AID transgene, together with hTau, and mouse are indicated in the panels. Selected samples are representative of the analysis conducted on all AID/hTau transgenics, at all ages. We found no significant difference in the amount and pattern of distribution of total Tau and its phosphorylations, in hippocampal and peri-hippocampal neuronal cellularity and microglia activation between controls and AID expressing mice.

Our biochemical data does not reveal a net trend of Tau phosphorylation induced by AID overexpression. Nonetheless, we wanted to ascertain if subtle changes could be seen at the tissue level, in both the quantity and the distribution of Tau phosphorylation. Furthermore, we have searched if any neuronal loss would be evident and if any microglia activation would be induced by AID overexpression. In Fig. 3, we show representative hippocampal immunostaining with antibodies against total Tau (DA9; Fig. 3A, B, C), pSer202 (CP13; Fig. 3D, E, F), pSer396/404 (PHF1; Fig. 3G, H, I). We could not detect any substantial difference in the amount and pattern of Tau phosphorylation, in the hippocampal area and parahippocampal cortex, attributable to AID overexpression. When using NeuN (Fig. 3J, K) and Iba1 (Fig. 3L, M) antibodies, neither neuronal loss nor microglia activation was seen. We did not perform unbiased stereological neuronal counting [39], given the paucity of indications toward a substantial loss of neurons in these mice. Microglia staining did not show signs of either recruiting or activation [40]. Of note, these mice show Tau distribution and phosphorylation similar to what previously described [29].

### GSK3α/β is not significantly upregulated by AID overexpression

**Figure 4:**
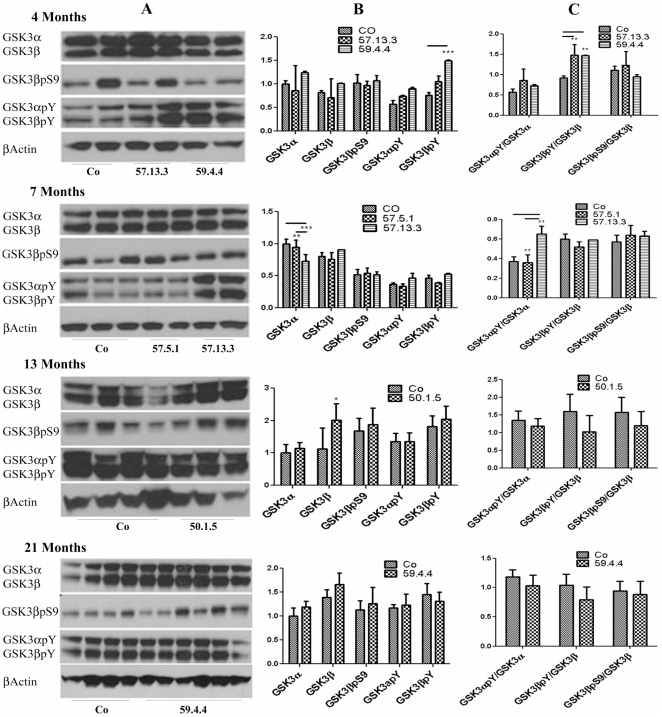
Western Blot analysis of GSK3β activation. Forebrain lysates from different lines of AID/hTau mice, at 4 different ages, were run on PAGE (Column A) and probed with antibodies against total GSK3α and β, the inhibiting phosphorylation pSer9 and the activating phosphorylation pTyrα279/β216. Densitometric quantification over β-Actin only (Column B) and over total GSK3α/β (Column C), shows no clear cut activation or inhibition of GSK3βby AID overexpression, related neither to AID length, mouse line nor age. Experiments were repeated at least 3 times on at least 2 mice/line. Quantifications units are arbitrary. *p<0.05; **p<0.01; ***P<0.001.

Although the data presented do not hint to a role for AID in promoting Tau accumulation or deranged phosphorylation, it is still possible that transgenic AID may cause hyperphosphorylation of Tau at later time, by activating kinases. Another model of AID59 overexpression [22], [28] shows GSK3β activation as early as 4 months, heterogeneous Tau phosphorylation as early as 8 months, and neurodegeneration at 18 months. When we analyzed our transgenic mice however, we could not detect any significant and consistent activation of GSK3β (Fig. 4, column C). The AID 57 and 59 transgenic mice show some GSK3β activation at 4 months, which is however not seen in older mice.

## Discussion

To directly examine the effects of AID *in vivo*, in the CNS, we generated transgenic mice expressing CaMKIIα-*AID*, to target AID expression to areas that are most relevant to Alzheimer's pathology [32]. We generated transgenic lines expressing either the 59- 57- or 50-residue AID peptide, which would be produced by γ-cleavage together with either Aβ40 or Aβ42, respectively, or the “ε-cleavage” [41], [42]. The AID50 is reputed the naturally occurring AID fragment. We obtained two AID59 (AID59-4.4 and -1.1), four AID57 (AID57-13.3, -5.1, -5.2 and -8.1) and three AID50 (AID50-3.4, -1.5 and 5.2) founder mice. Some of these lines have been analyzed here (AID50.1.5, AID57 5.1 and 13.3, AID59.4.4). When transgenic mice are used, it is important to study lines derived from more then one founder. This precaution is necessary to avoid erroneously attributing a phenotype, caused by an insertional effect on endogenous mouse genes, to the transgene itself.

A previous analysis of these mice showed that overexpression of AID did not affect gene transcription. However, cultured neuronal cells derived from AID transgenic mice were more sensitive to selective apoptotic and toxic stimuli. This evidence suggests that AID overexpression may predispose to neuronal degeneration. Thus, we tested whether *in vivo* AID expression could initiate aspects of AD that precede and lead to neuronal loss.

Contrarily to previous reports however [22], [28], we failed to see an AID-dependent activation of GSK3β and the phosphorylation of Tau phosphorylation typical of AD. What could the basis of these discrepancies be? There are several possibilities, which do not need to be mutually exclusive. The findings implicating AID in GSK3β activation and Tau pathology are based on the use of a single mouse line, and the double transgenic model expresses an AID molecule (AID 59) that probably does not correspond to the one produced *in vivo* by processing of APP-CTFs. This is problematic, for the reasons explained above. Although some evidence pointed to a role of AID as a “nuclear regulator” per se [43], the animal model used in these studies expresses Fe65, an APP binding protein, together with AID 59, [44]. Thus, the effectual contribution of AID to the described phenotypes is not obviously clear. Mice transgenic for AID alone would have helped, in that specific setting, to clarify the relative contribution of Fe65 and the intracellular domain of APP to the observed phenotypes. AID is very short lived [12] and it has been argued that Fe65 could potentiate AID functions by stabilizing it [45]. On the other side, it has been suggested that AID, recruiting Fe65 to the plasma membrane, is then able to release it and allow its transcriptional function to take part in the nucleus, with or without Tip60 [11], [17], [46]. The relationships between AID, Fe65, transcriptional control and induction of apoptosis are still uncertain, but several facts are of note. AID overexpression in our model is very high, as determined by Real Time PCR, and as evident from WB analysis [27]. In particular, we have shown how AID 57 and 59 are well expressed at the protein level, while AID 50 is not, although its mRNA is abundant. Such overexpression, we can assume, would probably be sufficient to activate kinases and promote Tau phosphorylation, even in the absence of Fe65 overexpression.

Do our data exclude a role for AID in AD? A common view for mechanisms underlying early pathophysiology of Alzheimer's disease (AD) includes axonopathy [47] and changes at synaptic level leading to subtle amnesic symptoms at an early stage of disease [48]. APP processing occurring in axonal extensions and/or synapses, leading to regional production of AID, may play a physiological role that, if disregulated, could lead to synaptic disfunction. In transgenic animals, most of AID is produced in the cell body, “far” from those neuronal compartments (axons/synapses) were this peptide might play its pathophysiological role. It is also likely that reduction in AID levels plays a pathogenic role in AD. The evidence that mutations in *PSEN1/PSEN2* that cause Familial Alzheimer's disease are loss of function mutants, and that loss of *PSEN1/PSEN2* function causes neurodegeneration in mice, supports this hypothesis [49], [50], [51], [52], [53]. In these cases, AD pathology coincides with lower production of total Aβ, an increase of the Aβ42/Aβ40 ratio, and a reduction of total AID.

In conclusion, our transgenic model shows that overexpression of several isoforms of AID in the CNS fails to reproduce obvious signs of AD-like pathology in mice. These data question whether a generalized overexpression of this APP fragment can address its role in both biology and disease if it is disjointed from the *in vivo* physiopathological context in which it is generated. Given these premises, AID and its functions should be investigated not much as a source of AD pathology, but as a possible interesting intracellular signaling pathway, much similar to NOTCH and NICD, that is still obscure but highly likely to be effective in neurons, possibly under stressing conditions.
